# Role of endoscopic endonasal approach for craniopharyngiomas extending into the third ventricle in adults

**DOI:** 10.1016/j.bas.2022.100910

**Published:** 2022-06-30

**Authors:** Matteo Zoli, Federica Guaraldi, Corrado Zenesini, Nicola Acciarri, Giacomo Sollini, Sofia Asioli, Marco Faustini-Fustini, Raffaele Agati, Luigi Cirillo, Caterina Tonon, Raffaele Lodi, Ernesto Pasquini, Diego Mazzatenta

**Affiliations:** a IRCCS Istituto delle Scienze Neurologiche di Bologna, Programma Neurochirurgia Ipofisi - Pituitary Unit, Bologna, Italy; b Department of Biomedical and Neuromotor Sciences, University of Bologna, Italy; c IRCCS Istituto delle Scienze Neurologiche di Bologna, Epidemiology and Statistics Unit, Bologna, Italy; d Azienda USL di Bologna, Anatomic Pathology Unit, Bellaria Hospital, Bologna, Italy; e Azienda USL di Bologna, ENT Department, Bellaria Hospital, Bologna, Italy; f IRCCS Istituto delle Scienze Neurologiche di Bologna, Programma Neuroradiologia con Tecniche ad Elevata Complessità, Bologna, Italy; g IRCCS Istituto delle Scienze Neurologiche di Bologna, UOC di Neuroradiologia, Bologna, Italy; h IRCCS Istituto delle Scienze Neurologiche di Bologna, Programma Neuroimmagini Funzionali e Molecolari, Bologna, Italy; i IRCCS Istituto delle Scienze Neurologiche di Bologna, Bologna, Italy

## Abstract

• EAA is an innovative, promising, safe and effective approach for 3VCPs.
• Key of success is surgeon learning curve in endoscopy and patients selection.
• With correct indications, EEA gives GTR and morbidity rate similar to other routes.
• Clinical, tumoral and anatomical features should be considered for EEA selection.

## 1 Introduction

Surgery of craniopharyngiomas extending into the third ventricle (3VCPs) is challenging for the risk of severe and permanent clinical sequelae, and even of fatal complications (Ahmed et al., 2018; Almeida et al., 2020; Apuzzo et al., 1982, 1991; Hardesty et al., 2018). In the last years, the endoscopic endonasal approach (EEA) has been proposed for the management of 3CVPs to reduce patient morbidity and improve surgical outcomes (Hardesty et al., 2018; Cagnazzo et al., 2018; Chamoun and Couldwell, 2013; Coppens and Couldwell, 2010; Forbes et al., 2018; Jean, 2018; Liu, 2013; Shukla, 2015; Silva et al., 2013; Zieli ń ski et al., 2018).

EEA was initially indicated only for the treatment of tumors with no or limited supra-diaphragmatic extension. Subsequent studies have progressively demonstrated its effectiveness and safety even for larger tumors with a suprasellar expansion (Hardesty et al., 2018; Cavallo et al., 2014; Cossu et al., 2020; Todeschini et al., 2018). Indeed, systematic reviews and meta-analyses have demonstrated a significant reduction of neurological sequelae (i.e., cranial nerves palsies, post-operative seizures, neurological deficits) together with the improvement of the visual outcome associated with EEA, thanks to the limited brain retraction and artero-nervous structures manipulation (Cavallo et al., 2014; Cossu et al., 2020; Todeschini et al., 2018; Algattas et al., 2020). However, EEA still presents some limitations, as, the difficulty in managing non-purely midline lesions and tumors encasing the main arteries and is burdened by a not negligible rate of post-operative CSF leak, with the consequent risk of post-operative meningitis (Almeida et al., 2020; Cavallo et al., 2014; Cossu et al., 2020; Todeschini et al., 2018; Algattas et al., 2020). Nowadays, only few studies have investigated the outcomes of EEA for 3VCPs, and despite their encouraging results, the role of EEA its selection for 3VCPs and criteria remain to be ascertained (Cavallo et al., 2014; Algattas et al., 2020; Koutourousiou et al., 2018; Leng et al., 2012; Fomichev et al., 2016).

Aims of this study were to assess the outcome of EEA in a large series of 3VCPs; defining its advantages and limitations; and to identify the factors favoring the choice of this approach, based on which we proposed a flow-chart to guide the approach selection for 3VCPs.

## 2 Materials and methods

Records of all consecutive adult patients (age >18 years old) affected by craniopharyngiomas, treated at the Pituitary Unit of the IRCCS Istituto delle Scienze Neurologiche di Bologna (Italy) from January 1998 to December 2020, were retrospectively analyzed to identify those extending to the 3rd ventricle (Kassam grade IV) and operated via EEA (Kassam et al., 2008).

Our protocol adopted for patient management has been previously reported (Foschi et al., 2017; Mazzatenta et al., 2020; Zoli et al., 2014, 2016). Briefly, in all patients, basal pituitary function, serum and urinary electrolytes and main anthropometric parameters, as BMI, were assessed preoperatively. All patients also underwent an ophthalmological (including visual field) and neurological evaluation, a MRI with gadolinium contrast medium, and a CT scan with angiographic sequences (CTA).

Pre-operative MRI was used to define the tumor relationship with the hypothalamus, differentiating the tubero-infundibular forms (characterized by circumferential displacement of the hypothalamus around the tumor), from the intra-ventricular forms, (with inferior displacement of the floor of 3rd ventricle), according to the classification proposed by Pascual et al. (Prieto et al., 2018, 2022; Pascual et al., 2013). The direction of displacement of the optic nerves and chiasm by the tumor was also analyzed. Finally, the neoplasm maximum diameter and consistency (i.e., cystic, mixed solid-cystic, or solid) and the presence of hydrocephalus were assessed. Tumor relationship with the internal carotid and other major vessels, and the presence of calcifications were defined by CTA.

Data on surgical technique, early and late surgical outcome, intra- and post-operative complications were collected from medical records. Endocrinological, neurological and ophthalmological evaluations, and MRI with contrast medium, were repeated at 3-month follow-up, then annually.

Tumor resection was considered ‘gross total’ (GTR) in case of no visible remnants at post-operative MRI, otherwise ‘partial’ (PTR). ‘Recurrence’ was defined as tumor detection in a patient with previously negative MRI, while ‘tumor progression’ indicated tumor growth at subsequent MRI studies. According to clinical and MRI features, patients with tumor recurrence or progression could be treated conservatively, or undergo second surgery, adjuvant radiation and/or medical treatment with BRAF/MEK pathways inhibitors (Chik et al., 2021; Marucci et al., 2015).

Patient quality of life was evaluated at follow-ups using the Katz index of independence in activities of daily living (Table 1) (Katz et al., 1963).

**Table 1:** Criteria for the definition of the level of functional patient outcome (adapted from Katz et al., *JAMA*, 1963) (Katz et al., 1963).

Level	Functional Outcome
**1**	complete autonomy in daily activities and social and at work/scholar tasks
**2**	partial autonomy in daily activities and social and work/scholar tasks
**3**	occasional external support necessary for daily life and impossibility to fulfill any social and work/scholar tasks, i.e. a semi-dependence condition
**4**	daily life absolutely dependent from continuous external support, i.e. a condition of absolute dependence

### 2.1 Surgical technique

The patient was placed in a semi-sitting position, with the thorax slightly elevated (20°). Surgery was performed under general anesthesia with oro-tracheal intubation. Laryngopharynx was packed with gauzes to prevent blood leakage in the upper respiratory tract. Rod lens endoscopes (SPIES, Karl Storz, Tuttlingen, Germany, 4 ​mm in diameter, 18 ​cm in length, with 0° and 30° scopes) with a high-definition camera were adopted. Neuronavigation (StealthStation S8 MEDTRONIC, Louisville, CO. USA), based CTA and MRI, processed through StealthMerge Software (MEDTRONIC, Louisville, CO. USA) was adopted in patients with lack of pneumatization or conchal variants of the sphenoidal sinus, and in second surgeries. A monolateral middle turbinectomy was initially performed. Currently, we prefer to avoid this maneuver to preserve middle nasal turbinates. Afterwards, a posterior septostomy was performed to operate through both nostrils, together with an anterior wide sphenoidotomy. In patients with a wide sphenoid pneumatization, we avoided ethmoidectomy.

After the identification of the anatomical landmarks of the posterior wall of sphenoid sinus, the sellar and the tuberculum bone were removed up to the sphenoidal planum with a high-speed diamond drill and/or a Kerrison rongeur. The superior intercavernous sinus was coagulated using bipolar forceps. Then, the dura was cut to access to the supradiaphragmatic region. At this point, the endoscope was fixed by a holder to allow tumor resection with a “four hands/two nostrils” technique.

Tumor was progressively localized and removed through central debulking followed by its cleavage from the surrounding arachnoid layer. During subarachnoid dissection, extreme care was paid to avoid injury to optic structures and preserve the superior hypophyseal artery. Cystic portions of the tumor were marsupialized, then their wall was removed as much extensively as possible. The preservation of the pituitary stalk was tried if it was not infiltrated by the tumor. Finally, the tumor was progressively dissected from the hypothalamus. In case of subpial infiltration, resection was stopped to avoid permanent neurological sequelae. Special attention was paid to preserve the posterior part of the hypothalamus and the mammillary bodies. After the completion of tumor removal, the surgical cavity was explored using an angled scope to detect potential tumor remnants, or active bleeding.

Plastic repair of the osteo-dural opening was performed with dural substitute (Biodesign, Cook Medical, Bloomington, IN, USA), fascia lata, fat, bone and mucoperiosteum graft, or naso-septal flap. Before 2013, we preferentially adopted a multilayer or gasket seal technique that included fascia lata, fat, (eventually bone) and a free graft of mucoperiostium; later, it was replaced by a multilayer technique performed with a dural substitute, fat (eventually bone) and a pedicled flap. Sphenoid sinus was packed with gelfoam, and a single Merocel (Merocel Corp., Mystic, CT) was inserted in both nostrils.

### 2.2 Statistical analysis

Pre-operative clinical and neuroradiological data of patients with 3VCPs operated via EEA were compared with those of patients treated by other surgical approaches (i.e., transcranial, TCA, or endoscopic trans-ventricular, ETA), to identify predictors of positive outcome associated with each approach.

Continue variables were presented as mean ​± ​Standard Deviation (SD), while categorical variables as absolute (n) and relative frequency (%). Kruskal Wallis test for continuous variables and Chi-square test or Fisher's exact test for categorical variables were used to evaluate the association between pre-operative features of interest (i.e., age; gender; BMI; previous treatments; endocrinological, visual and neurological status; relationship between tumor and hypothalamus; presence of hydrocephalus; optic chiasm displacement; tumor size; tumor calcification and consistency) with surgical outcome for the different surgical approaches. Significant associations were combined in a flow-chart aimed at guiding the choice of the most suitable surgical approach.

Statistical analysis was performed using STATA (Statistical software, Version 13 – StataCorp LP. College Station, Texas).

## 3 Results

**Fig. 1:**
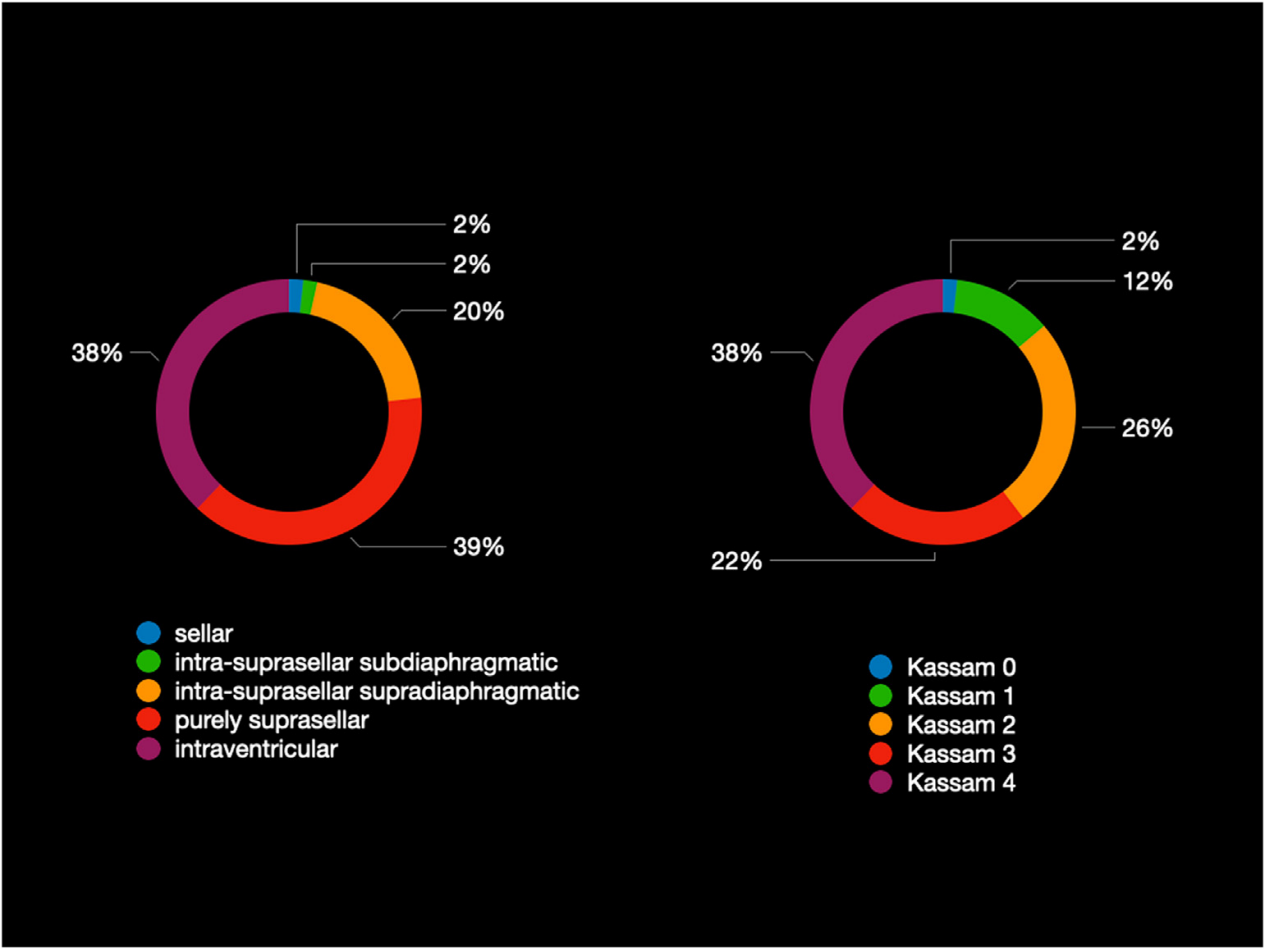
Distribution of craniopharyngiomas extending into the 3rd ventricle (3VCPs) with respect to the 116 adult patients with craniopharyngiomas operated at our Center from 1998 to 2020.

EEA was adopted in 36 out of 50 patients with 3VCPs; the remnant cases underwent TCA (n ​= ​10) or ETA (n ​= ​4) (Fig. 1). Clinical and radiological features are reported in Table 2, Table 3.

**Table 2:** Patient demographic and clinical features at surgery.

	Total (n ​= ​50)	EEA (n ​= ​36)	TCA (n ​= ​10)	ETA (n ​= ​4)	p
**Females (N, %)**	25 (50.0)	19 (52.8)	4 (40.0)	2 (50.0)	0.86
**Age** (years; mean ​± ​SD)	54.3 ​± ​15.5	51.1 ​± ​15.9	63.3 ​± ​7.6	60.5 ​± ​18.6	**0.04**
**Age>70 years (N, %)**	7 (14.0)	3 (8.3)	2 (20)	2 (50)	**0.02**
**BMI** (kg/m^2^; mean ​± ​SD)	27.4 ​± ​5.4	27.9 ​± ​6.0	26.2 ​± ​3.2	25.1 ​± ​2.9	0.63
**Previous treatments (N, %)**					0.52
None	44 (88.0)	32 (88.8)	9 (90.0)	3 (75.0)	
Surgery (craniotomy)	4 (8.0)	2 (5.6)	1 (10.0)	1 (25.0)	
Surgery and radiation therapy	2 (4.0)	2 (5.6)	0 (0)	0 (0)	
**Endocrinological Alterations (N, %)**					**0.05**
None	19 (48.0)	10 (27.8)	7 (70.0)	2 (50.0)	
Partial anterior hypopituitarism	15 (30.0)	14 (38.8)	1 (10.0)	0 (0)	
Panhypopituitarism	1 (2.0)	1 (2.8)	0 (0)	0 (0)	
DI	2 (4.0)	2 (5.6)	0 (0)	0 (0)	
Partial hypopituitarism and DI	4 (8.0)	4 (11.1)	0 (0)	0 (0)	
Panhypopituitarism with DI	9 (18.0)	5 (13.9)	2 (20.0)	2 (50.0)	
**Visual Acuity Deficits (N, %)**					0.48
No	37 (74)	25 (69.4)	8 (80.0)	4 (100.0)	
Yes	13 (26.0)	11 (30.6)	2 (20.0)	0 (0)	
**Visual Field Deficits (N, %)**					**<0.01**
None	14 (28.0)	6 (16.7)	7 (70.0)	1 (25.0)	
Bilateral quadrantopia	7 (14.0)	6 (16.7)	1 (10.0)	0 (0)	
Bitemporal hemianopia	22 (44.0)	18 (49.9)	1 (10.0)	3 (75.0)	
Bilateral involvement of >2 quadrants	7 (14.0)	6 (16.7)	1 (10.0)	0 (0)	
**Neurological Symptoms (N, %)**					**<0.01**
None	39 (78.0)	31 (86.1)	5 (50.0)	3 (75.0)	
Ophtalmoplegia	1 (2.0)	0 (0)	1 (10.0)	0 (0)	
Intracranial hypertension	10 (20.0)	5 (13.9)	4 (40.0)	1 (25.0)	

Legend to table: DI ​= ​diabetes insipidus; EEA ​= ​endoscopic endonasal approach; ETA ​= ​endoscopic transventricular approach; N ​= ​number of patients; NS: not significant; SD: standard deviation; TCA ​= ​transcranial approach (p values ​< ​0.05 are reported in bold).

**Table 3:** Neuroradiological and tumoral features.

	Total (N ​= ​50)	EEA (N ​= ​36)	TCA (N ​= ​10)	ETA (N ​= ​4)	p
**Maximum Tumor Diameter** (mm; mean ​± ​SD)	26 ​± ​10	28 ​± ​10	21 ​± ​6	17 ​± ​9	0.60
**Hydrocephalus** (N, %)	15 (30.0)	6 (16.7)	5 (50.0)	4 (100)	**<0.01**
**Tumor Location** (N, %)					
Tubero-infundibular	41 (82.0)	36 (100.0)	2 (20.0)	3 (75.0)	**<0.01**
Intra-ventricular	9 (20.0)	0 (0)	8 (80.0)	1 (25.0)	**<0.01**
**Chiasm Displacement** (N, %)					
Antero-superior	38 (76.0)	36 (100.0)	1 (10.0)	1 (25.0)	**<0.01**
Antero-inferior	12 (24.0)	0 (0.0)	9 (90.0)	3 (75.0)	**<0.01**
**Calcifications** (N, %)	34 (68.0)	24 (66.7)	8 (80.0)	0 (0)	0.06
Egg-shell shape	30 (60.0)	23 (63.8)	7 (70.0)	0 (0)	
Nodular shape	2 (4.0)	1 (2.8)	1 (10.0)	0 (0)	
**Consistency**					
Cystic	6 (12.0)	1 (2.8)	1 (10.0)	4 (100)	**0.05**
Mixed (solid and cystic)	29 (58.0)	23 (63.9)	6 (60.0)	0 (0)	**0.05**
Solid	15 (30.0)	12 (33.3)	3 (30.0)	0 (0)	**0.05**

Legend to table: EEA ​= ​endoscopic endonasal approach; ETA ​= ​endoscopic transventricular approach; N ​= ​number of patients; NS: not significant; SD: standard deviation; TCA ​= ​transcranial approach (p values ​< ​0.05 are reported in bold).

### 3.1 Pre-operative assessment

Nineteen patients were females (52.8%); mean age at surgery was 51.1 ​± ​15.9 years (Table 2). Mean BMI at hospital admittance was of 27.9 ​± ​6.0 ​kg/m^2^. Thirty-two patients (88.8%) were naive for treatment, 4 (11.1%) had already undergone TCA, followed by radiotherapy in 2 cases (5.6%).

Pituitary function was normal in 10 patients (27.8%); 15 (41.6%) presented with anterior hypopituitarism, 2 (5.6%) with isolated DI and 9 (25%) with anterior hypopituitarism and DI. Visual disturbances were the most common presenting symptom; 11 patients (30.6%) reported the reduction of visual acuity, while 30 (83.3%) had campimetric deficits. Five patients (13.9%) were admitted with manifestations (i.e., headache, nausea, vomiting, initial consciousness impairment) suggestive for intracranial hypertension.

Mean tumor diameter was of 28 ​± ​10 ​mm. All tumors were classified as tubero-infundibular forms, with antero-superior displacement of the optic chiasm. MRI demonstrated the presence of obstructive hydrocephalus in 6 patients (16.7%) (Table 3). At CT-scan, 24 (66.6%) tumors presented calcifications. Twenty-three tumors (63.9%) were of solid-cystic consistency.

### 3.2 Surgical outcome and complications

An extended transplanum-transtuberculum approach was performed in all cases. Histologically, 12 (33.3%) cases were papillary while 24 (66.7%) were adamantinomatous forms.

GTR was achieved in 33 patients (91.7%) (Table 4).

**Table 4:** Surgical outcome, complications and follow-up.

	EEA (N ​= ​36)	TCA (N ​= ​10)	ETA (N ​= ​4)
**Radical Resection**	33 (91.7)	7 (70.0)	0 (0)
**Complications**			
CSF Leak	5 (13.9)	1 (10.0)	0 (0)
Hematoma	1 (2.8)	2 (20.0)	0 (0)
Hydrocephalus	0 (0)	3 (30.0)	0 (0)
Seizures	0 (0)	1 (10.0)	0 (0)
Transitory Memory Loss	1 (2.8)	0 (0)	0 (0)
Epistaxis	1 (2.8)	0 (0)	0 (0)
III CN palsy	1 (2.8) Transient	1 (10.0) Permanent	0 (0)
**Quality of life at follow-up**			
Level 1	32 (88.8)	3 (30.0)	1 (25.0)
Level 2	2 (5.6)	2 (20.0)	2 (50.0)
Level 3	0 (0)	0 (0)	0 (0)
Level 4	1 (2.8)	1 (10.0)	0 (0)
Deceased	1 (2.8)	4 (40.0)	1 (25.0)
**Long-Term Follow-up**			
Progression	2 (5.6)	2 (20.0)	4 (100.0)
Progression time (months; mean ​± ​SD)	22 ​± ​13	38 ​± ​23	18 ​± ​5
Treatment for tumor progression (N,type)	1 TCA ​+ ​RT 1 RT	1 TCA 1 RT	1 TCA 1 ETCD ​+ ​TCA 1 ETCD 1 WS
Recurrence	6 (16.7)	1 (10.0)	0 (0)
Recurrence time (months; mean ​± ​SD)	35 ​± ​29	30	/
Treatment for tumor recurrence (N, type)	2 TCA 1 EEA 1 RT 1 RT ​+ ​CMT 1 WS	1 TCA ​+ ​RT	/

Legend to table: CMT: chemotherapy; CN ​= ​cranial nerve; CSF: cerebor-spinal fluid; EEA ​= ​endoscopic endonasal approach; ETCD ​= ​endoscopic transventricular cyst drainage; N ​= ​number of patients; RT ​= ​radiation therapy; TCA ​= ​transcranial approach; WS ​= ​wait and see.

The most frequent complication was post-operative CSF leak, overall occurring in 5 cases (13.9%) −3 among the 14 patients (21.4%) operated before 2013, and 2 among the 22 (9.1%) treated later -, that were all promptly re-operated via EEA. Nevertheless, 3 patients developed meningitis (8.3%), that was treated with i.v. antibiotic therapy, with no neurological sequelae.

One patient (2.8%) presented with sudden loss of consciousness in the first day after surgery, due to a 3rd ventricle hematoma. An external ventricular drainage was immediately inserted to treat hydrocephalus, followed by EEA surgery to remove the clots and decompress the surrounding neural structures. In the following days, the patient recovered consciousness and the external ventricular drainage was removed. At last follow-up, she presented with no focal neurological deficits, but remained dependent for daily life activities.

Another patient developed post-operative epistaxis due to the bleeding of a branch of the spheno-palatine artery, requiring re-intervention for cauterization.

Finally, one patient presented with transitory palsy of the 3rd cranial nerve, and another with transitory memory disturbance, that fully and spontaneously recovered after 3 and 6 months, respectively.

### 3.3 Follow-up

Mean follow-up was of 43 ​± ​38 months. At last evaluation, BMI was 29.4 ​± ​6.5 ​kg/m^2^ (Δ BMI: 1.49 ​± ​3.59).

Thirty-five patients (97.2%) presented with panhypopituitarism with DI (Table 5). Visual acuity improved in 6/11 cases with pre-operative deficits (54.5%),and campimetric deficits in 21/30 (70%). Deterioration of the visual field and acuity occurred in 2 patients (5.5%). In the remaining cases, visual acuity and field remained stable at follow-up. Intracranial hypertension resolved in all cases. One patient (2.8%) died 88 months from surgery for causes unrelated to tumor or surgery.

**Table 5:** Post-operative clinical outcome.

	Last follow-up			
Pre-operative	**Stability (n)**	**Improvement (n)**	**Normalization (n)**	**Worsening (n)**
**EEA**				
**Endocrinological alteration**				
None	0	0	0	10
Partial hypopituitarism	0	0	0	14
Panhypopituitarism	0	0	0	1
DI	0	0	0	2
Partial hypopituitarism ​+ ​DI	1	0	0	3
Panhypopituitarism ​+ ​DI	5	0	0	0
**Visual Acuity Deficit**				
No	23	0	0	2
Yes	5	6	0	0
**Visual Field Deficit**				
None	6	0	0	0
Bilateral quadrantopia	1	2	2	1
Bitemporal hemianopia	4	10	3	1
Bilateral involvement of >2 quadrants	2	4	0	0
**TCA**				
**Endocrinological alteration**				
None	2	0	0	5
Partial hypopituitarism	0	0	0	1
Panhypopituitarism	0	0	0	0
DI	0	0	0	0
Partial hypopituitarism ​+ ​DI	0	0	0	0
Panhypopituitarism ​+ ​DI	2	0	0	0
**Visual Acuity Deficit**				
No	5	0	0	1
Yes	1	1	0	0
**Visual Field Deficit**				
None	6	0	0	1
Bilateral quadrantopia	0	1	0	0
Bitemporal hemianopia	0	1	0	0
Bilateral involvement of >2 quadrants	0	1	0	0
**ETA**				
**Endocrinological alteration**				
None	2	0	0	0
Partial hypopituitarism	0	0	0	0
Panhypopituitarism	0	0	0	0
DI	0	0	0	0
Partial hypopituitarism ​+ ​DI	0	0	0	0
Panhypopituitarism ​+ ​DI	2	0	0	0
**Visual Acuity Deficit**				
No	4	0	0	0
Yes	0	0	0	0
**Visual Field Deficit**				
None	1	0	0	0
Bilateral quadrantopia	0	0	0	0
Bitemporal hemianopia	0	1	2	0
Bilateral involvement of >2 quadrants	0	0	0	0

Legend to table: DI ​= ​diabetes insipidus; EEA ​= ​endoscopic endonasal approach; N ​= ​number of patients

Quality of life (QoL) was preserved in 32 (88.8%) cases, with return to pre-operative daily and professional life (level 1 of Katz Index). Two cases (5.6%) presented with partial post-operative functional recovery (level 2), while one (2.8%) remained completely dependent from caregivers for daily activities (level 4) (Table 4).

Tumor progression was observed in 2 patients (5.6%) after a mean of 22 ​± ​13 months. One was treated with proton beam therapy, the other with TCA surgery followed by photon radiation therapy. Six patients (16.7%) presented with tumor recurrence after a mean of 35 ​± ​29 months; 2 were treated with TCA, 1 with EEA, 1 with photon radiation therapy, and another with proton therapy followed by chemotherapy with BRAF/MEK pathway inhibitors for multiple disseminations through the CSF. A conservative approach, with periodic clinical and MRI monitoring, was chosen in a patient with an asymptomatic sub-centimetric cystic recurrence (Table 4).

### 3.4 Approach selection analysis

According to statistical analysis, significant differences between patients treated with EEA and other approaches were observed in the following parameter: tumor relationship with the hypothalamus, direction of optic chiasm displacement, presence of hydrocephalus, tumor cystic consistency, patient age, endocrinological, visual and neurological status.

Specifically, EEA was preferred in tubero-infundibular tumors with antero-superior optic chiasm displacement, while TCA in intra-ventricular forms with inferior chiasm displacement (p ​< ​0.01). Patients with hydrocephalus were preferentially treated with TCA and ETCD (p ​< ​0.01), while cystic consistency favored ETA (p ​= ​0.05). EEA and TCA were preferred in younger patients, and ETA in the elderly (p ​= ​0.04). In patients with intact hypothalamic-pituitary function TCA and ETA were preferred to EEA (p ​= ​0.05). Finally, EEA was preferred in case of severe visual field deficit, and TCA in patients with intracranial hypertension (p ​< ​0.01).

**Fig. 2:**
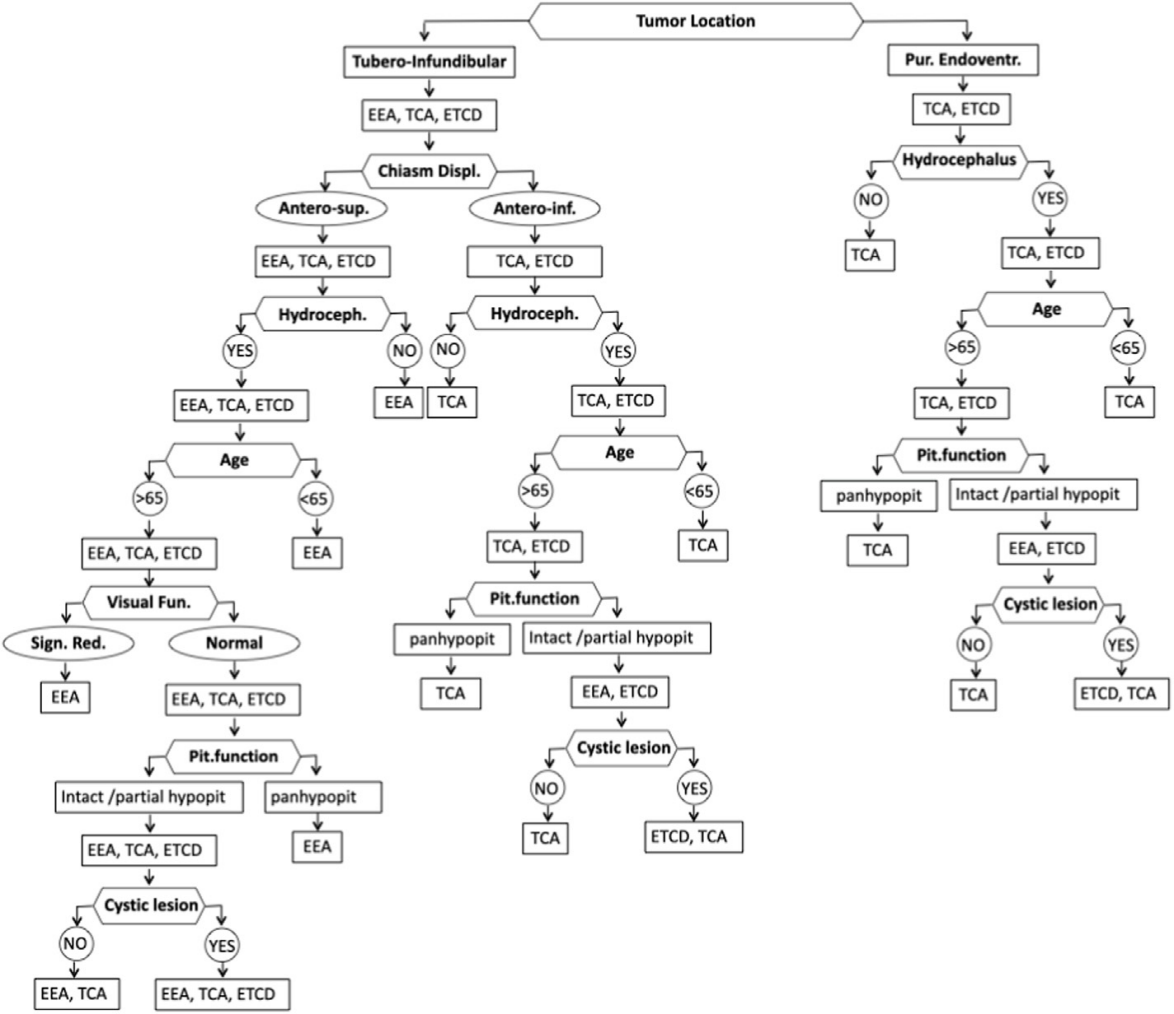
Descriptive flow-chart for treatment selection (EEA ​= ​endoscopic endonasal approach; ETA ​= ​endoscopic transventricular approach; TCA ​= ​transcranial approach, pit.:pituitary, hypopit.: hypopituitarism).

A flow-chart was created based on study findings to guide the choice of surgical approach in patients with 3VCPs (Fig. 2). The significance of the following variables was confirmed at subsequent model elaboration and were, therefore, combined to create the flow-chart: tumor relationship with the hypothalamus, direction of chiasm displacement, presence of hydrocephalus, patient age at surgery, pre-operative visual and endocrinological status, and tumor consistency.

### 3.5 Case illustration

**Fig. 3:**
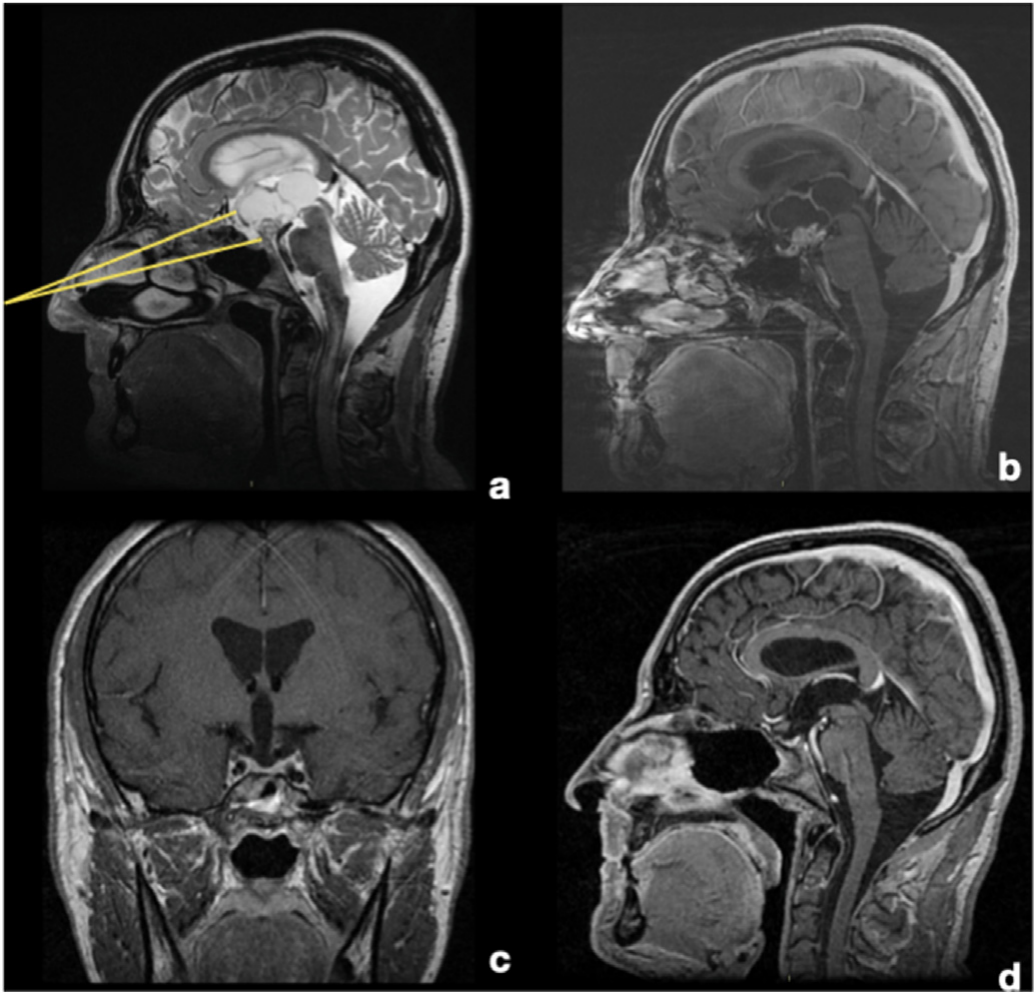
MRI T2-w and T1-w with gadolinium (sagittal and coronal slices). A and B. Pre-operative MRI demonstrating a partially cystic tumor invading the third ventricle. The suprasellar portion is mostly solid with some calcifications, while the intra-ventricular one has a mixed consistency, with a large cystic appearance. Pituitary stalk is not clearly recognizable, but it seems displaced posteriorly by the tumor Hypothalamic structures were displaced circumferentially around the tumor, while the optic chiasm was displaced antero-superiorly and no signs of hydrocephalus were present. The EEA was chosen because, as showed with yellow lines, it could give a direct and straight corridor to the tumor, without the interposition of any eloquent structures, as optic chiasm, stalk or hypothalamus. C and D. Post-operative MRI. A complete tumor removal has been achieved. The optic chiasm has been decompressed and it has recovered its normal location, while hypothalamic structures have been preserved. Because of the strict adherence between the tumor and the stalk, it has been resected during surgery. (For interpretation of the references to colour in this figure legend, the reader is referred to the Web version of this article.)

A 47-year-old male was admitted to the emergency room for a rapid worsening of the visual acuity and narrowing of the visual field. Ophthalmological evaluation demonstrated a visual acuity of 3/10 in the right eye, and of 1/10 in left eye, together with bitemporal hemianopia. Endocrinological investigations showed central hypocortisolism (8 a.m. serum cortisol 57 ​ng/ml; ACTH 12 ​pg/ml), hypogonadism (LH 1.4 mUI/ml; FSH 3.3. mUI/ml; total testosterone 1.9 ​ng/ml) and hypothyroidism (TSH 1.56 mcU/ml; fT4 6.8 ​pg/ml), hyperprolactinemia (35 ​ng/ml), and central DI. Pre-operative BMI was 26.5 ​kg/m^2^. MRI showed suprasellar solid/cystic lesion, located in the tubero-infundibular junction, with extensive invasion of the 3rd ventricle and antero-superior displacement of the chiasm, without hydrocephalus (Fig. 3).

After collegial discussion, surgery via EEA was chosen. The tumor was initially centrally debulked, and then dissected from the surrounding structures, using the arachnoid as cleavage plane, following its extension into the third ventricle. Pituitary stalk could not be preserved because of the firm tumor adherence. Plastic repair was performed with a multilayer technique using fascia lata, fat, a naso-septal flap, and a piece of septal bone. Histological diagnosis was of adamantinomatous craniopharyngioma.

The post-operative course was unremarkable. At follow-up (69 months), the patient presented with panhypopituitarism with central DI. The BMI was 28.9 ​kg/m^2^ (5-kg weight increase). Visual field impairment had partially restored, while no improvement of acuity deficit was observed. The MRI demonstrated a radical tumor resection (Fig. 3).

## 4 Discussion

In the last years, the pendulum of the goal of the treatment of both adults and pediatric craniopharyngiomas has moved from radical resection toward maximal safe resection, eventually followed by adjuvant therapies (i.e., radiation or pharmacological treatments), being the primary aim the preservation of patient's QoL (Hardesty et al., 2018; Mazzatenta et al., 2020; Konovalov, 2014; Asha et al., 2020). This change has important implications in case of 3VCPs, indeed because of their close anatomical relationship with many eloquent neural and vascular structures, their surgery is high risk of permanent endocrinological, ophthalmological, neurological and neurocognitive sequelae (Mazzatenta et al., 2020; Konovalov, 2014; Asha et al., 2020).

Various surgical approaches are available for 3VCPs, including TCAs (i.e. transcallosal, frontotemporal or “pterional” craniotomies, “mini-pterional”, lateral supraorbital, frontotemporal orbital-zygomatic and fronto-orbital approaches via eyelid or eyebrow incisions), that have significantly evolved in the last years to miniaturize their extension, thus reducing the brain retraction as much as possible (Ahmed et al., 2018; Jean, 2018; Liu, 2013; Shukla, 2015; Silva et al., 2013; Zieli ń ski et al., 2018; Cai et al., 2019; Ndukuba et al., 2020; Shoubash et al., 2022; Delitala et al., 2004; Cinalli et al., 2006; Labidi et al., 2018). The implementation of the endoscopic-assisted technique, that consists in the use of the endoscope for the entire surgery or for a part of it through a small transcranial access, has effectively contributed to reduce the invasiveness of these approaches (Ahmed et al., 2018; Jean, 2018; Liu, 2013; Shukla, 2015; Silva et al., 2013; Zieli ń ski et al., 2018; Cai et al., 2019; Ndukuba et al., 2020; Shoubash et al., 2022; Delitala et al., 2004; Cinalli et al., 2006; Labidi et al., 2018). Moreover, neuroendoscopic transventricular approaches allow the surgeon to drain the tumor cysts, decompressing the neural structures and restoring CSF flow in case of obstructive hydrocephalus through a single burr-hole, with limited risk of complications (Ahmed et al., 2018; Jean, 2018; Liu, 2013; Shukla, 2015; Silva et al., 2013; Zieli ń ski et al., 2018; Cai et al., 2019; Ndukuba et al., 2020; Shoubash et al., 2022; Delitala et al., 2004; Cinalli et al., 2006; Labidi et al., 2018). EEA has been proposed only recently for 3VCPs, with the first pioneeristic cases dating back only to the early 2010s (Cavallo et al., 2014; Leng et al., 2012; de Lara et al., 2013). Also in our series, the vast majority of 3VCPs (33; 91.7%) have been operated by EEA in the last decade, after a long experience with this technique, that had started in 1998 ​at our Center.

The rate of GTR obtained by EEA in our series was high (91.7%) and similar (82%) to that reported by a recent literature review on the outcomes of TCA (Algattas et al., 2020). In our hands, EEA also appeared effective in preserving QoL (in (88.9%), also with a limited increase in mean BMI at follow-up (Δ: 1.49 ​± ​3.59). To achieve these results, we consider of paramount importance the optimal visualization of the interface between the tumor and the hypothalamus allowed by the endoscope, and the straightforwardness associated with the endonasal approach. These elements allowed us to stop the tumor resection in case of hypothalamic infiltration, avoiding its direct injury, particularly to the posterior structures, as of the mammillary bodies. However, we consider that not all 3VCPs are suitable for EEA, but should be reserved to carefully selected cases.

### 4.1 Patients selection

Currently, no specific recommendations have been reported to guide surgeons in the EEA selection for 3VCPs (Hardesty et al., 2018). Our study allowed the identification of some important anatomical, tumor and clinical features, which should be kept in consideration in the patients selection for an EEA.

**Fig. 4:**
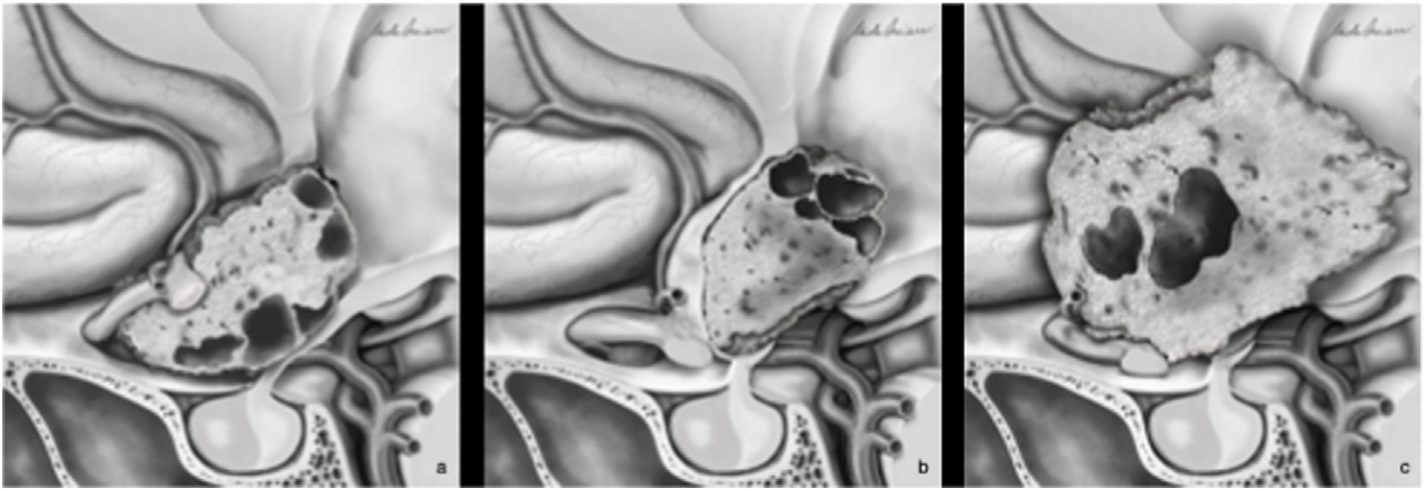
Schematic drawnings representing the anatomical conditions favoring or discouraging the EEA for 3VCPs. A. The tubero-indibular origin of the tumor and the antero-superior displacement of the chiasm create a straight and safe corridor, allowing the surgeon to approach the tumor along its growth axis with an EEA. B. The purely intra-ventricular location of the 3VCPs, with the hypothalamic structures displaced antero-inferiorly represent a limitation for EEA, for the interposition of these eloquent structures along the surgical corridor, increasing the risk of direct or indirect injuries, with consequent functional sequelae and possible reduction of patient QoL. C. A tubero-infudibular location, but with antero-inferior displacement of the chiasm represents a further limitation for EEA.

In particular, our study confirmed that purely intra-ventricular forms represent an absolute contra-indication to EEA. Therefore, tumor extension is the first parameter that has to be considered in the decision-making process, reserving the EEA to the tubero-infundibular forms (Fig. 4). The second element to be evaluated is the direction of chiasm displacement. The antero-superior displacement creates a large and straight corridor that can be exploited to reach the neoplasms via EEA, limiting the risk of chiasm injury during surgical maneuvers (Fig. 4) (Zoli et al., 2014). Conversely, in case of antero-inferior chiasm displacement, the optic structures are interposed along the endoscopic endonasal route, with a significant risk of surgical damage; on the other hand, the working space through the trans-lamina terminalis is consequently increased, thus supporting the choice of TCA (Fig. 4) (Omay et al., 2018; Kim et al., 2018).

Whenever allowed by the anatomical features, EEA should be preferred in younger patients presenting with visual and endocrinological deficits, without hydrocephalus. In our experience, stalk location, nor the tumor size represented a limit to EEA, and we observed even large tumors could be safely removed, if all other selection criteria were respected.

### 4.2 Endocrinological outcome

According to previous literature, the endocrinological outcome of surgical treatment of 3VCPs is usually poor. Indeed, these tumors can infiltrate the hypothalamic centers that regulates pituitary function, and the stalk, with consequent common pre-operative anterior and posterior pituitary insufficiency (Cavallo et al., 2014; Locatelli et al., 2018; Solari et al., 2016).

An open question in the management of 3VCPs is whether the respect of the anatomical integrity of the stalk could increase the chances of preserving the hypothalamic-pituitary function, and whether this advantage counterbalances the increased risk of tumor recurrence (Forbes et al., 2018; Ord óñ ez-Rubiano et al., 2018). Ordonez-Rubiano et al. demonstrated that the preservation of the stalk reduced the risk of post-operative pituitary deficit of about 50%, but was counterbalanced by a significant increase of the risk of tumor progression requiring irradiation, as previously argued by Jung et al. (Ord óñ ez-Rubiano et al., 2018; Jung et al., 2009; Albano et al., 2020). Therefore, stalk management remains debated. (Algattas et al., 2020). We attempted to preserve the stalk whenever possible, except for cases with evident tumor infiltration, but in case of evident tumor infiltration, we preferred its resection to achieve a more radical resection and reduce the risk of recurrences.

Recent studies performed on large cohorts showed that the prevalence of post-operative hypopituitarism and DI, is similar in patients treated with EEA and TCA (Algattas et al., 2020). This underlines that 3VCPs surgery is burdened by a high risk of long-term pituitary functions, independently from the adopted approach.

### 4.3 Visual outcome

We observed an improvement in 54.5% of cases with visual acuity deficits, and of 70% in those with campimetric alterations, that are in line with the results reported by previous studies (Cavallo et al., 2014; Algattas et al., 2020; Locatelli et al., 2018; Solari et al., 2016). Algattas et al. reported an improvement/normalization of visual symptoms in 74% of patients with 3VCPs treated by EEA, with a rate significantly superior to one achieved by TCA (45%) (Cavallo et al., 2014; Algattas et al., 2020; Locatelli et al., 2018; Solari et al., 2016). The better outcomes associated with EEA could derive from the lack of manipulation of the optic chiasm and the sparing of its vascularization, given by the subarachnoidal dissection of the tumor, performed when approaching the 3VCP through this ventral route (Cavallo et al., 2014; Locatelli et al., 2018; Solari et al., 2016). However, it is important to remark that these good results depend also by the surgeon experience and strict patients selection.

### 4.4 Complications management

Most of the surgical complications consisted of post-operative CSF leak (13.9%), that mainly occurred in the first years of our surgical experience with 3VCPs, in particular before the introduction in 2013 of the naso-septal flap (21.4 vs. 9.1%). This supports the importance of introduction of the pedicled flap and of the perfectioning of the plastic repair techniques in promoting the adoption of EEA for 3VCPs (Cavallo et al., 2019). Indeed, the high rate of post-operative CSF reported by early studies had significantly limited the widespread of EEA to treat 3VCPs in the last years. However, in case of post-operative CSF leak we suggest an aggressive management, with quick detection and an early surgical revision of the plastic repair, to avoid the development of a post-operative meningitis (Milanese et al., 2017).

Moreover, EEA was associated with a limited risk of developing post-operative hydrocephalus in 3VCPs, indeed in our series no permanent ventricular shunts have been necessary (Algattas et al., 2020). It remains to be clarified if this depends on the 3rd ventricle opening into the basal cisterns related to EEA, or if it is a bias related to the lower number of craniopharyngiomas presenting with pre-operative hydrocephalus in this patient series (Algattas et al., 2020).

Despite the apparent optimal safety profile, it is fundamental to remark that this approach for 3VCPs requires a long training curve, and should, therefore, be reserved to dedicated Centers, to reduce the risk of dramatic surgical complications.

### 4.5 Study limitations

The main study limitation is represented by the retrospective study design, that could have hampered the collection of other parameters, potentially relevant in the choice of the surgical approach. Moreover, even if ours is one of the larger series in literature, the rarity of these tumors led to the collection of a limited number of patients, possibly reducing the statical power. The wide timeframe considered can be represent a bias, for the technical advancements occurred during that period. Finally, a direct comparison of the outcome of the different techniques was not possible for the unavoidable selection bias.

## 5 Conclusions

3VCPs are rare and complex tumors, which can be effectively and safely resected with an EEA in selected cases. This approach has the advantage of a straight and direct extracranial route toward the tumor, which avoids any brain manipulation or retraction. However, it cannot be considered as ideal for all 3VCPs and a strict and careful patients selection, based on anatomical, clinical and tumor features of each single case is needed.

Based on our experience, EEA should be preferred in tubero-infubular forms with antero-superior displacement of the chiasm, especially in younger patients with visual and/or endocrinological deficits and without hydrocephalus.

However, this approach needs a long and dedicated training and it should be reserved to selected specialized centers. Finally, further studies are strongly advised to better profile surgical indications, towards a more personalized and successful treatment.

## Conflict of interest

The authors state that the content of the submitting manuscript, in part or in full, has not been published previously and has not been submitted elsewhere for review.

There is no financial support received in conjunction with the generation of this submission and no conflict of interest.

The Authors have nothing to declare and nothing to disclose and no conflict of interest that could be perceived as prejudicing the impartiality of the research reported.

## Disclosure and conflicts of interest

The authors state that the content of the submitting manuscript, in part or in full, has not been published previously and has not been submitted elsewhere for review.

There is no financial support received in conjunction with the generation of this submission and no conflict of interest.

The Authors certify that this manuscript is a unique submission and is not being considered for publication with any other source in any medium.

The Authors have nothing to declare and nothing to disclose and no conflict of interest that could be perceived as prejudicing the impartiality of the research reported.
